# Potential of Mesenchymal Stem Cells in the Rejuvenation of the Aging Immune System

**DOI:** 10.3390/ijms22115749

**Published:** 2021-05-27

**Authors:** Genieve Ee Chia Yeo, Min Hwei Ng, Fazlina Binti Nordin, Jia Xian Law

**Affiliations:** Centre for Tissue Engineering and Regenerative Medicine, Faculty of Medicine, Universiti Kebangsaan Malaysia Medical Centre, Jalan Yaacob Latif, Cheras 56000, Malaysia; genieve.yec@gmail.com (G.E.C.Y.); angela@ppukm.ukm.edu.my (M.H.N.); nordinf@ppukm.ukm.edu.my (F.B.N.)

**Keywords:** mesenchymal stem cells, aging, inflammaging, frailty, immune system

## Abstract

Rapid growth of the geriatric population has been made possible with advancements in pharmaceutical and health sciences. Hence, age-associated diseases are becoming more common. Aging encompasses deterioration of the immune system, known as immunosenescence. Dysregulation of the immune cell production, differentiation, and functioning lead to a chronic subclinical inflammatory state termed inflammaging. The hallmarks of the aging immune system are decreased naïve cells, increased memory cells, and increased serum levels of pro-inflammatory cytokines. Mesenchymal stem cell (MSC) transplantation is a promising solution to halt immunosenescence as the cells have excellent immunomodulatory functions and low immunogenicity. This review compiles the present knowledge of the causes and changes of the aging immune system and the potential of MSC transplantation as a regenerative therapy for immunosenescence.

## 1. Introduction

Frailty can be defined as a decline in physiological reserve across organ systems; it afflicts geriatric subjects above the age of 65. Immunosenescence is the term used to refer to profound changes in the immune system related to age. Immunosenescence involves dysregulation of the immune functions at both cellular and serological levels [1]. As a result of degenerating immunity, older age groups are more susceptible to severe infections with poor prognoses [2]. The risk of contracting community-acquired pneumonia increased by 21% in older adults of 65–74 years compared to younger patients, with an even higher incidence in older adults 85 years and above [3,4]. Bacteremia and sepsis are also more prevalent among older adults. More troubling—older adults have a higher risk of morbidity and developing cognitive decline post-infection [5,6]. Interestingly, older adults have higher autoimmunity, but not autoimmune disorders. The autoimmunity in older adults is associated with the high levels of circulating T-regulatory cells (Treg) and reduced CD4/CD8 ratio. Subsequently, this predisposes the aging host to infection and cancer [7]. The increasing age also hampers the effects of vaccination unless the vaccine is developed to bypass this concern, i.e., conjugating it with an adjuvant. Hence, immunization for the aging population is limited [1,8,9,10].

As a direct result of senescence, the immune system is in a constant subclinical inflammatory state known as ‘inflammaging’. Inflammaging is conjectured to be a consequence of activation of innate immunity and declination of adaptive immunity without exogenous stimuli. This state is associated with the cytokine’s milieu that skews towards a pro-inflammatory phenotype. The exact relationship between inflammaging and the disease state is yet to be elucidated [11]. However, most age-related degenerative diseases share similar inflammatory pathogenesis to which inflammaging may further exacerbate the disease process and its morbidity. The common inflammatory disease includes cardiovascular disease (myocardial infarction, hypertension, atherosclerosis), cognitive impairments (Alzheimer’s disease, Parkinson’s disease), rheumatoid arthritis, and metabolic diseases (type II diabetes) [11,12,13].

The population aging is rapidly accelerating. The United Nations speculates that the number of people aged 65 years old and older will double between 2019 and 2050. In 30 years, one out of six people worldwide will be categorized in the “older adult” age bracket [14]. The older adult population is accompanied by a state of physiological vulnerability and declining ability to maintain homeostasis and respond to stress. This clinical expression of age-related decline is also known as frailty. Frailty and inflammation are strongly correlated where the serum levels of inflammatory markers are significantly higher in the older age group compared to the younger age group [15]. Frailty includes functional and structural alterations in multiple organ systems and impaired immune responses, which predispose to a plethora of disorders [16,17]. Consequently, the older adult population is a significant financial burden to the healthcare system [18]. Although the presentation of diseases may be incited by other risk factors, aging is a significant contributing mechanism due to the inevitable frailty development.

Currently, there are a few measures that may delay frailty onset and improve the morbidity of age-associated disease. The management of geriatric patients includes implementing calorie restriction, exercise regimes, and hormonal supplementations [12,19,20]. Diets high in n-3 polyunsaturated fatty acids and vitamin D have positive outcomes in reducing circulating levels of inflammatory molecules, namely C-reactive protein (CRP) and interleukin (IL)-6, as well as lower the mortality of the inflammatory diseases [21,22]. Zhang et al. showed that physical exercise can delay cognitive impairment while Ng et al. reported that cognitive training can improve physical mobility and strength [23,24]. The studies also showed that a mix of interventions (exercise and/or nutrition and/or cognitive training) would have better results than just either one [25]. Frailty is a complex condition that is unique to every individual; these clinical treatments require personalization to directly intercept immunological frailty. Moreover, Zhang et al. have found that the frailty index scoring system does not necessarily reflect the conditions the subject is facing. Some elderly may still be classified as pre-frail due to the cut-off score, but were experiencing frailty in different domains, be it cognitive or functional [23]. In the systemic review composed by Apostolo et al., the current personalized approach to manage disease-associated frailty has failed to produce consistent results [25]. Hence, there is yet an exact solution to frailty.

Mesenchymal stem cells (MSCs) are multipotent progenitor cells that can be isolated from the bone marrow, adipose tissue, dental tissues, skin, salivary gland, limb buds, menstrual blood, and perinatal tissues [26,27,28,29]. MSCs can differentiate into adipocytes, osteoblasts, and chondrocytes. Although MSCs do not differentiate into immune cells, MSCs provide a supporting microenvironmental niche for hematopoietic stem cells (HSCs) to differentiate into myeloid and lymphoid cells, which are essentially the immune cells. This specialized environment plays an important role to maintain the longevity of HSCs by controlling their proliferation and apoptotic activities [30].

One of the speculated theories of declining immunity as the host ages is the MSC senescence. Subsequently, the functions and structures of MSCs, which are significant in maintaining the immune system, diminishes [31]. Although they are multipotent, mesenchymal progenitors exist in a small population, only consisting of 0.001% to 0.01% bone marrow mononuclear cells. Therefore, ex vivo expansion of MSCs and subsequent administration of optimized dosage is necessary to maintain and boost the effects of MSCs in vivo [32]. Furthermore, numerous in vivo and in vitro studies have proven that MSCs have low immunogenicity, excellent immunomodulatory function, and homing capability to regenerate damaged tissues through multipotent differentiation and paracrine secretion [11,33,34,35,36]. Despite that, the current studies are not primarily focused on aging or the restoration of the immune system. There have been extensive studies done on pathological conditions than actual aging itself. Aging and MSC were studied separately, but the similarities of the immune markers involved may come into convergence. The proliferative capacity and immunomodulatory function of MSCs could aid in the restoration of the immune cells and reduce the pro-inflammatory markers since these parameters are observed in aging as well. It is imperative to discuss the papers based on the aspects related to immunosenescence and inflammaging. 

This review aims to discuss the recent papers on the pathophysiology of immune system aging and the potential of MSC therapy to combat immunosenescence.

## 2. Causes and Consequence of Immunosenescence

There are several theories on the cause of immunosenescence. According to Lopez -Otin et al., there are eight hallmarks of aging. This includes genomic instability, telomere attrition, epigenetic alterations, loss of proteostasis, deregulated nutrient sensing, mitochondrial dysfunction, cellular senescence, stem cell exhaustion, and altered intercellular communication [37]. A review by Rodrigues et al. applied the hallmarks of aging to immunosenescence [38]. Few causes of immunosenescence that we are briefly introducing in this review include oxidative stress, mitochondrial reactive oxygen species (ROS), telomere attrition, thymic involution, impaired autophagy, epigenetic alterations, genomic instability, and cellular senescence. In general, the impact of immunosenescence on the structure, functions, and population of the immune cells is detrimental.

### 2.1. Oxidative Stress

Chronic oxidative inflammatory stress can lead to premature aging with immunosenescence. The essential components of the immune cells such as protein, lipids, and DNA are constantly damaged by oxidative stress, which diminishes their capacity to maintain redox and inflammatory balance. The incessant oxidative stress causes constant stimulation of the inflammasome, which induces the nuclear factor-κB (NF-κB) and the IL-1β-mediated inflammatory cascade. Additionally, the senescence-associated secretory phenotype (SASP) contributes to the constant subclinical inflammation by producing a self-perpetuating intracellular signaling loop [11]. Garrido et al. determined that the peritoneal leucocytes of both prematurely aged and chronologically aged mice have reduced levels of antioxidants (catalase and glutathione reductase activities), increased levels of oxidants (xanthine oxidase activity, oxidized glutathione levels, oxidized and reduced glutathione ratios), and increased secretion of pro-inflammatory cytokines (IL-1β, IL-6, and tumor necrosis factor (TNF)-α) without stimulation. Moreover, the same study observed that this oxidative-inflicted damage reduces the catecholamine concentration in the peritoneal macrophages, which is a key component in immunomodulation during stress response [39].

### 2.2. Mitochondrial ROS

In-line with oxidation-inflammaging stress, another causative theory of immunosenescence is accumulated mitochondrial oxidative stress. ROS is an inevitable by-product of oxidative phosphorylation and other biochemical processes. ROS is an essential component in the regulation of physiological cellular functions such as growth, proliferation, differentiation, and apoptosis. At low concentration, ROS is essential for a healthy immune response and to induce inflammation through the activation of leukocyte recruitment process. Pathogens can trigger a respiratory burst of ROS, which attracts neutrophils to form clusters. Then, ROS will resolve inflammation by inducing the apoptosis of neutrophils. However, in excess, ROS can be detrimental to the cellular proteins, RNA, and DNA. Naturally, it is one of the suspected culprits of immune system aging. With age, the body’s ability to maintain redox balance becomes impaired, leading to excessive ROS levels which cause oxidative stress in the mitochondria of immune cells [40]. T-memory cells (Tmem) and Treg rely highly on oxidative phosphorylation; they carry a large mitochondrial mass, which allows them to rapidly respond to their cognate antigens. Mitochondria also regulate calcium ions (Ca^2+^), which is pertinent to the activation of the immune signaling pathway that controls the activation of T cells. Along with increasing age, the increased mitochondrial mass and the dysregulation of membrane potential in the mitochondria of CD8+ T cells was noted by Sanderson and Simon [40]. Furthermore, at old age, ROS increases the level of plasma mitochondrial DNA (mtDNA) which is proportional to the levels of pro-inflammatory cytokines [41,42]. Besides, in vivo data from injecting mtDNA to induce inflammation and interfering with its inflammatory pathway shows that mtDNA, not nuclear DNA, is the contributor to inflammatory pathogenesis [41]. In general, the data implies that the degradation of mitochondria due to ROS increases with age and this leads to the dysfunction of adaptive immune response, subsequently causing inflammation.

### 2.3. Telomere Attrition

On the other hand, telomere shortening is depicted as a phenomenon of replicative stress which leads to senescence. The accumulated detrimental effects of persistent ROS, mitochondrial dysfunction and DNA damage at the telomeres force the cells to enter a transient proliferation arrest. The highly proliferative component of the immune system, the T cells are particularly susceptive to replicative stress and driven to senescence. Sanderson and Simon confirmed that telomere attrition is significantly correlated with age in CD8+ T cells, as the capacity to proliferate dwindled. However, the differences in relative telomere length versus age is insignificant in B cells [40]. Furthermore, naïve T cells from the older adults are found to have shorter telomeres than younger people, signifying a diminished capacity to proliferate. When T cells have prolonged interaction with antigen, they extensively proliferate which accelerates the loss of telomeric DNA. Subsequently, the shortened telomeres in the CD8+ T cell population lead to decreased vaccine efficacy in the older adults [1].

### 2.4. Thymic Involution

Next theory of immune system aging is thymic involution. The thymus is a primary lymphoid organ, which is essential to the adaptive immunity, whereby it is the place the T cells become mature. The thymus is non-self-renewing and must rely on the production of T cell progenitors or thymocytes in the bone marrow through hemopoiesis of HSCs. Around adolescence, the thymus begins to degenerate, resulting in a gradual loss of tissue mass and structure that progresses along with age. Production of IL-7, which is necessary for thymopoiesis, also reduces with age. This phenomenon of thymic involution diminishes the supporting microenvironment vital for the maturation of T cells, causing a decrease in the output of naïve CD4+ or CD8+ T cells [43,44,45]. Sidler et al. found alterations in the gene expression of cell cycle regulation of old rats, resulting in a large proportion of splenic and thymic cells with incomplete cell division [46]. The effect of senescent HSCs is also apparent in the early thymocyte progenitor (ETP) activity, in which T cell differentiation is reduced, apoptotic activity is heightened and Ki67+ cells are reduced [44,45,47]. Thymic involution is an inevitable process of aging but induced pluripotent stem cells (iPSCs) have shown promising results in the regeneration of thymic epithelium. Otsuka et al. demonstrated that mice iPSCs integrated with exogenous Foxn1 gene can successfully differentiate into thymic epithelial cells. This finding further develops the prospect of growing thymus grafts from iPSCs for transplantation [48].

### 2.5. Impaired Autophagy

Autophagy is a cellular catabolic process, which mediates the degradation of cellular components when fused with lysosomes. This mechanism provides an alternative source of energy for protein synthesis and to sustain metabolism during metabolic stress. Autophagy modulates both innate and adaptive immune responses. The innate and adaptive immune cells require autophagy to differentiate, activate, and function. Innate immune receptors stimulate pathogen removal through autophagy, whereas autophagy enhances the T cells’ antigen presentation step by speeding up the delivery of antigen to lysosomes. Autophagy also regulates the secretion of inflammatory cytokines by T cells, such as interferon gamma (IFN-γ). Moreover, autophagy suppresses inflammation through the degradation of ubiquitinated inflammasome [49,50]. The autophagy system is activated by intracellular and extracellular stress signals, such as oxidative stress. In old age, the compounded detrimental effects of oxidative stress produce a defective autophagy mechanism, in which the compromised protein degradation system has reduced capacity to remove the misfolded proteins and damaged macromolecules in the cells [11]. As a result, the maturation, activation, and antigen processing ability of immune cells are impaired [51].

### 2.6. Epigenetic Alteration

Epigenetic changes in aging involve histone modifications, DNA methylation, and chromatin remodeling. Histones undergo various post-translational modifications (PTMs), including acetylation, methylation and phosphorylation, which are reversible by specialized histone-modifying enzymes [52,53,54]. 

A study has shown that senescent fibroblast cells reduced histone biosynthesis, lysosomal-mediated processing, and increased macroH2A, leading to decreased histones. The level of macroH2A was elevated in the aged mice lungs and livers [55]. A study on the postovulatory aging of the mouse oocyte reported the gradual acetylation on some lysines of histones H3 and H4 [56]. Cheng et al.’s study in human and mouse brains found that there was a loss of acetylated-H3K27 during aging, along with the increase of enzyme histone deacetylase-2 (HDAC-2) activity, which contributed to cognitive decline. However, this phenomenon can be reversed by HDAC-inhibitor [57]. Treatment with HDAC-inhibitor have also successfully improved the DNA repair and extended the lifespan of the Zmpste24^−/−^ mice [58]. These findings show that some aging, which is caused by epigenetic influences, is reversible. 

After receiving pro-inflammatory signal, the acetylation of H4 and H3 occurs and leads to the increased recruitment of NF- κB. NF- κB is one of the important molecules in the inflammatory pathway as it promotes various cytokines and chemokines during inflammaging, along with the proinflammatory IL-6. Then, IL-6 regulates the DNA methyltransferases (Dnmt), which can be affected by ROS. Cao et al. determined that a DNA methyl transferase inhibitor, decitabine effectively reduced Dnmt activity and attenuated NF-κB activation [59].

Lastly, in response to DNA damage, the chromatin structure is remodeled by nucleosome to form senescence-associated heterochromatin foci (SAHF). Chromatin accessibility is also modulated by the exchange of histone variants. As a result, the transcription activity of proliferation-promoting genes is reduced and the gene loci are sequestered into the SAHF [58,60,61]. One of the chromatin remodeling mechanism is a non-histone chromatin-bound protein called high mobility group box 2 (HMGB2), which is involved in upregulating the SASP loci through the alteration of the chromatin architecture [60]. On the other hand, the HMGB1 relies on p53 to induce senescent growth arrest, which is different from the ataxia-telangiectasia mutated protein (ATM)-dependent SASP [62].

### 2.7. Genomic Instability

Genomic instability is another hallmark of aging and cancer. The instability is contributed by the persistent deoxyribonucleic acid (DNA) damage, defects in the nuclear lamina, and mutations in the mtDNA [37]. Throughout the lifespan of the living organism, genetic damage is accumulated from the DNA damage from both exogenous and endogenous source. The endogenous impact includes DNA replication errors, spontaneous hydrolytic reactions, and ROS. Cells may develop malignancy from these accumulated insults and form proteins called neoantigens, which they then can be identified from. These genome alterations may trigger T cell recognition and induce immune attack towards the malignant cells. The main components of DNA damage response are the early signal transducer, ATM and signal transducer for replicative stress, ataxia telangiectasia, and Rad3-related protein (ATR). Telomeres will be lost at every cell division and eventually become shortened as the organism ages. These shortened telomeres, alongside persistent DNA damage, lead to the accumulation of ATM and ATR effectors at the sites of damage. As a result, the DNA-SCARs (DNA segments with chromatin alterations reinforcing senescence) are formed [63].

Krishnan et al. described a recurrent point mutation in the lamin A gene, which accelerates aging in both humans and mice. The mutation that occurs at the G608G position impairs the processing of prelamin A into mature lamin A, and instead, forms the mutant protein, progerin [58]. Progerin causes nuclear blebbing, accumulation of DNA damage, and accelerated cellular senescence. Individuals with such mutation have the Hutchinson-Gilford Progeria Syndrome (HGPS), which is a rare disorder that resembles premature aging [64,65].

Aside from the nuclear DNA, genomic instability can also be seen in the mtDNA. The mtDNA is very prone to somatic mutations due to its lack of protective histones and limited repair mechanisms unlike in nuclear DNA. Multiple mtDNA exists in within the same cell with varying mutant and wild-type genomes. This is termed “heteroplasmy”. In aging cells, the mutational load becomes significant and “homoplasmy” is achieved due to the prevalence of a single mutant genome. Recently, Vizioli et al. discovered the formation of a SASP trigger called cytoplasmic chromatin fragments (CCFs) through activation of the innate immunity cytosolic DNA sensing cGAS–STING pathway. This pathway is targetable by drug interventions such as the HDAC-inhibitor. They reported that the compound effectively suppressed oxidative stress, CCDs and SASP, both in vitro and in vivo, and successfully restored the mitochondria function. The decreased mitophagy in the senescent mitochondria can also be restored by the HDAC-inhibitor [66,67].

### 2.8. Cellular Senescence

In a normal condition, senescent cells do occur, but they are eliminated by immune cells and replaced. However, in aging condition, the senescent cells are not removed efficiently and tend to accumulate over time. SASP is a unique feature of senescent cells which induce autocrine and paracrine signaling of pro-inflammatory factors. SASP consists of molecules that are commonly upregulated in inflammaging such as IL-6, TGF-β, IL-1α, TNF-α, matrix metalloproteinases (MMPs), insulin-like growth factor-1 binding proteins (IGFBPs), and other non-soluble extracellular matrix proteins. Senescence-associated β-galactosidase (SA-β-Gal) is a hallmark of senescent cell, in which its expression can be modulated by single-nucleotide polymorphism-1 (SIRT1). SASP occurs in response only to persistent DNA damage signaling and is dependent upon the DNA damage response (DDR) proteins ATM, Nijmegen breakage syndrome 1 (NBS1), and checkpoint kinase 2 (CHK2). The NF-κB pathway is also activated by the DDR proteins [68,69].

Even without the presence of DDR, senescence can still be formed from the overexpression of the cyclin-dependent kinase inhibitors CDKN1a/p21^Cip1^ or CDKN2a/p16^INK4A^ [70,71]. The reduced expression of p16^INK4a^, which can be identified by a diminished SA-β-gal activity can ameliorate an age-related decline in T cell responsiveness to CD3 and CD28 [71,72,73]. Moreover, the transcriptional activation of the CDKN2a locus prevents the proteasomal degradation of p53 through Mdm2 inactivation using p14^ARF^ [74]. p53 is another aging factor as its overexpression has been shown to induce premature senescence in mice in multiple tissue types [75,76,77]. The senescence-inducing capacity of p53 has potential in treating various cancers and aging T cells replicative senescence [78,79].

SASP is both the result of aging and the driver of further senescence. According to Ogata et al., the senescent fibroblasts are usually cleared by the induced apoptosis by TNF-α secreted from macrophages, then the phosphatidylserine (PS) receptors would be exposed and recognized by the macrophages for phagocytosis. Nevertheless, in aging, this SASP phenotype impairs the clearance of senescent cells by attenuating the function of the immune cells, and also precipitate an accumulation of aged cells that exceeds the immune cells clearance capacity [80]. 

## 3. Age-Associated Changes in the Innate Immune System

The age-associated changes in the innate immunity are relatively milder than seen in the adaptive immunity. Nevertheless, notable differences can be made of the innate immune cells between the young and old subjects. Animal and human studies have demonstrated that aged HSCs appear to exhibit an increased bias toward myeloid differentiation with a reduced capacity toward lymphoid differentiation [44,81,82]. Ergen et al. stated that the inflammatory cytokine, RANTES, which is elevated with aging, is responsible in stimulating the myeloid biased HSCs and diminishing the lymphoid output [82]. In frail subjects, the myeloid cells including monocytes and natural killer (NK) cell counts are elevated. Neutrophil count is reported to be constant or elevated [83,84,85,86]. Moreover, there are reported changes in the innate immune cells function including reduced chemotaxis, diminished phagocytic capacity, increased pro-inflammatory cytokine expression and altered signaling pathways in response to antigens and granulocyte colony-stimulating factor (G-CSF) [83,87].

### 3.1. Monocytes/Macrophages 

One of the significant alterations in the innate immunity with age is the upregulated expression of inflammatory pathway genes in monocytes/macrophages including the pro-inflammatory marker IL-6 [17]. An in vitro study by Hsieh et al. observed the effect of senescence on dengue virus infection. The monocytes which were induced into senescence using D-galactose exhibit pro-inflammatory activity and increased DC-SIGN (CD209) expression, which indicates an increased propensity to viral, bacterial, and parasitic infection. The increase in DC-SIGN is partially attributed by the higher secretion of IL-10 by senescence monocytes [2].

On the other hand, the aging non-classical monocytes actively secrete excessive levels of TNF-α and IL-8 [86]. In the older adults, the decreasing level of magnesium superoxide dismutase (MnSOD) is correlated with the increasing oxidative stress in the macrophage. *MnSOD* is an antioxidant enzyme located in the macrophage mitochondria matrix, which functions to protect the macrophages from low density lipoprotein (LDL)-induced apoptosis [87]. The toll-like receptors (TLRs), which act like a bridge between the innate and adaptive immune system declines with age. This results in an altered cytokine production and response which then affects the adaptive immune system [88,89,90].

Transforming growth factor (TGF)-β is another cytokine upregulated by senescent monocytes. TGF-β together with IL-10 suppress dendritic cell (DC) function and promote the M2-type macrophage polarization. Moreover, TGF-β level affects the adaptive immune system by converting naïve CD4+ T cells into Tregs, regulating the differentiation of T-helper type 1 (Th1) and Th2 cells, and inhibiting B cell proliferation and responsiveness [81,91]. Naturally, the dysregulated TGF-β secretion is detrimental to the upkeep of T and B cells as well. Consequently, the chronic age-related stimulation of monocytes in the absence of immunological insult leads to inflammaging.

### 3.2. Neutrophils

The neutrophil count throughout a person’s lifespan is relatively constant but some studies noted a decrease in function [92]. Wenisch et al. stated that the phagocytic capacity of neutrophils is impaired with age. Their study suggested that the neutrophils of the elderly have increased intracellular calcium concentrations at a resting state, decreased phagocytic ability, and diminished bactericidal activity due to the reduced production of intracellular ROS [93].

Furthermore, older adults are more prone to neutropenia during infection due to insensitivity to G-CSF. According to Zhang et al., the neutrophils are persistently activated in the aged microbiota through TLR and myeloid differentiation factor 88 (MyD88)-mediated signaling pathways. The neutrophils also have significantly elevated activation of TLR and NOD-like receptor (NLR), and NF-kB signaling pathways and express higher levels of TLR4 surface antigen [84]. Next, Roy-O’Reilly et al. stated that aging augments the ROS production in circulating neutrophils and suppresses the neutrophil clearance mechanism which results in an overabundance of circulating neutrophils [94]. Under normal conditions, the circulating neutrophils will be cleared in the bone marrow, liver, and spleen. However, the aged neutrophils proceed to accumulate at the site of inflammation. 

Unlike the other reports of neutrophils with diminished function due to age, Uhl et al. reported the age-related enhancement of the phagocytic capacity of the aged neutrophils through contracting the b2integrin Mac-1/CD11b and spleen tyrosine kinase-dependent signaling event. Uhl et al. also noted that aged neutrophils migrate more efficiently to the site of inflammation as they can instantly translate inflammatory signals to engage TLR-4 and p-38 MAPK-dependent pathway. Interestingly, the aged neutrophils did not have elevated respiratory burst nor cytokine production, which prevented the harmful effects to the surrounding tissue [95]. On the contrary, Zhang et al. mentioned that aged neutrophils tend to produce neutrophil extracellular traps (NETs) and ROS excessively, which will cause harm to the surrounding tissue [84].

All in all, the defective neutrophil function and their upregulated inflammatory activity may reduce the efficacy of the aging immune system in eliminating foreign pathogens, subsequently exacerbate disease outcomes. Nevertheless, the study by Uhl et al. suggested otherwise, which prompted more in-depth studies on the role of neutrophils in aging.

### 3.3. Natural Killer (NK) Cells

There is an increase in late NK cells as the host ages. However, this increase does not indicate an upregulated function, but simply an accumulation of long-standing NK cells [85,86,96]. The antiviral capacity decreases with age due to the decreased chromatin accessibility of their activating receptor [97].

## 4. Age-Associated Changes in the Adaptive Immune System

The adaptive immunity includes cell-mediated immunity and humoral immunity mediated by the T cells and B cells, respectively. The distinctive traits of adaptive immunosenescence include the decline of naïve lymphocytes and increasing antigen-experienced lymphocytes, especially of the memory phenotype. The senescent lymphocytes have limited capacity in eliminating novel antigens, have a pro-inflammatory cytokine profile, favors the development of autoimmunity and can evade apoptosis [86,98,99,100,101].

### 4.1. T Cells

The modifications in the adaptive immune compartment due to age largely compromises the immune responses and predisposes the older adults to frailty. The major alteration of the immune system is focused on the T cell repertoire. Under normal circumstances, T cells are central in the clearance of infection and tumor through immune-mediated cell death. The remodeling includes a population shift from naïve cells to terminally differentiated memory cells. The incessant replication of T cells in response to stimulate also exhausts the proliferation capacity, leading to senescence. In other words, as the subject gets older, his immunity enters an immune cell refractory state where the responses of both T and B cells to novel antigens decline [47,98,102]. The reduced CD4/CD8 ratio with age also indicates a higher risk of infections. The persistent antigen load, for example, the chronic cytomegalovirus (CMV) infections, which precipitated with age may be the cause of the expansion of both CD4+ and CD8+ Tmem, but in the cost of diversity [81,98,103]. These changes resulted in increased cytokine production, diminished chromatin remodeling, and poorer antiviral capacity [86].

On the contrary, Lelic et al. argued the CD8+ Tmem function is not age-dependent, and the responses to de novo viral antigens are comparable to young human subjects. The apparent decrease of naïve CD8+ T cells in the peripheral blood is not a full representative of the naïve T cell pool as naïve T cells may still be concentrated in the human lymphoid tissues. Nevertheless, the measurement of T cells collected in the tissues is not feasible for a living individual, and most data have been collected from murine models instead [98]. Even so, murine data may not be completely representative for human T cells. Xie et al. reported that old C57BL/6 mice (21 months old) did not show loss of CD28 expression but instead, they present a notable increase of CD28+ CD8+ T cells when compared to young mice (7 months old) [104]. To simulate the human immune system, humanized mice are developed by transplanting human CD34+ HSCs to immunodeficient mice. Nonetheless, the mice fail to demonstrate antigen-specific T effector cell response due to the maturation location in mice thymic environment [105].

CD28 is a costimulatory molecule to CD8+ T cells, which binds to CD80 on antigen-presenting cells to induce IL-2 to promote cellular survival and proliferation. The aging cells losing CD28 yet gaining CD57 is another characteristic of immunosenescence, which reflects its diminished capacity to proliferate [16,98,103]. Lee et al. also noted that stimulated CD28- T cells produce larger amounts of pro-inflammatory IFN-γ and TNF-α [99]. Senescent T cells that lost the CD28 expression also show resistance to apoptosis and diminished caspase 3 activity in response to apoptotic stimuli. Hence, these aged cells tend to accumulate and are irremovable by programmed cell death [85].

Next, the expansion of Treg cells aided by T-helper 17 cells (Th17) may precipitate autoimmunity in the older adults. Treg cells also dulls the CD4 and CD8 functions, which increases one’s susceptibility to infection and cancers [88,106].

Moreover, mitochondria within T cells play an essential role in regulating the secondary messengers especially Ca^2+^ and ROS. Mitochondria dysfunction is apparent with age, in which the defects are reflected in the diminished Ca^2+^ signaling in the T cells. In addition to that, oxidative phosphorylation (OXPHOS) and glycolysis are the main sources of energy in the T cells, with OXPHOS particularly essential to naïve T cells prior to activation and rapid proliferation. During an immune response, the T cells activated by TCR stimulation and CD28 switches from OXPHOS to glycolysis to satisfy their metabolic requirement. The reducing mitochondrial mass as result from active proliferation also favors the metabolic bias to glycolysis. Nevertheless, all T cell subsets still utilize OXPHOS but at a varying and generally reduced capacity [107,108]. Sanderson and Simon noted that CD8+ Tmem cells have increased mitochondrial mass in the older population, but the other T and B cells remain unchanged [40].

### 4.2. B Cells

The humoral component of the adaptive immune system, the B cells are not an exception to the immune remodeling caused by age. The characteristics of B cells in older adults include decreased production of high-affinity antibodies and diminished antibody responses to pathogens [86,101]. The aging pro-B cells have diminished ability to respond to IL-7, a hematopoietic growth factor essential to the maturation of B cells [100,101]. Then, the pre-B cells receptors which are lost due to the diminution of the surrogate light chain (SLC) also limits the expansion of pre-B cells. Consequently, only a proportion of naïve B cells mature into functional B cells [100,109].

The age-related defects on the B cell receptors reduce the affinity and signaling required to activate the B cells in response to stimuli. The mechanisms required to generate effective high affinity antibodies are compromised as shown in the decreased activation-induced cytidine deaminase (AID) expression, which is essential for somatic hypermutation and class-switch recombination. Moreover, the germinal center, which is essential for antibodies to undergo affinity maturation and somatic hypermutation, declines with age [47,100]. As demonstrated in the murine model, the immunization results in a similar amount of antibody but the affinity is severely reduced.

The prolonged elevation of circulating TNF-α level leads to the increment of TNF-α level within B cells. Additionally, the old follicular B cells also have higher secretion of TNF-α. This causes the formation of a larger proportion of exhausted B cells and decreased switched memory B cells. High level of endogenous TNF-α also deteriorates the antibody responses of B cells [100,102]. In addition, IL-21 and IFN-γ are found to promote the formation of aged B cells [47,100]. The ability of older adults to respond to de novo antigens is diminished due to the decrease in B cell repertoire diversity. This encompasses the loss of naïve B cells and the accumulation of long-lived memory cells in the B cell pool. The B cell receptor clonality also increased with age, indicating the decrease of unique clonotypes in B cells [86]. The diminished B cell functions may be related to the overexpression of SASP marker in the switched memory B cells in the older adults [110,111,112]. In spite of that, the memory cells produced in early life remain functional [101,113].

The age-associated B cells that gradually accumulate with age are more likely to secrete autoantibodies. In addition, B cells from older adults have poorer production of IL-10 that has been reported to reduce autoantibody production. Furthermore, the aged B cells tend to shift activated CD4+ T cells to Th17 phenotype, which is associated with autoimmune disorders. Thus, the B cells in the older adults become prone to causing autoimmune responses and may precipitate the development of autoimmune diseases [88,100,114]. The effects of age on the immune cells along with the prospects of MSC to improve those shortcomings are summarized in Table 1.

## 5. Mesenchymal Stem Cell Therapy to Reverse Aging Effects on the Immune System

According to The International Society for Cellular Therapy (ISCT) 2006, there are three minimal criteria for defining MSCs: “(i) MSC must be plastic-adherent when maintained in standard culture conditions. (ii) ≥95% of the MSC population must express CD105, CD73 and CD90, and ≤2% of the MSC population express of CD45, CD34, CD14 or CD11b, CD79a or CD19 and HLA Class II surface molecules. (iii) MSC must differentiate to osteoblasts, adipocytes and chondroblasts in vitro.” [141].

MSCs lack MHC class II, which translates to low immunogenicity and allows both allogeneic and autologous MSC transplantation. Standardization and commercialization of allogeneic MSCs would be readily available at a reduced cost due to the lack of need to create a personalized cell therapy from the autologous source and the potential of expanding the cells in large-scale [142]. To date, MSCs have been used in clinical trials for osteoarthritis, spinal cord injury, diabetes mellitus, autoimmune disease (Crohn’s disease, multiple sclerosis, systemic lupus erythematosus, and systemic sclerosis), and systemic diseases such as graft-versus-host diseases and sepsis [30,143,144,145]. Additionally, MSCs also have been used to treat neonatal diseases, i.e., intraventricular hemorrhage, bronchopulmonary dysplasia, and necrotizing enterocolitis [146].

### 5.1. Mechanism of MSCs Action on Immune System

Some evidences showed that the ameliorating effects of MSCs on the immune system are not due to direct engraftment and cell replacement, but rather paracrine manner and direct cell-to-cell contact [26,147]. MSCs secrete soluble paracrine factors including TGF-β, prostaglandin E2 (PGE2), indoleamine 2,3-dioxygenase (IDO), hepatocyte growth factor (HGF), nitric oxide (NO), interferon-gamma (IFN-γ), IL-2, and IL-10, which produce an immunomodulatory effect. They also express FasL and PD-L1 for contact-dependent inhibition to induce T cell apoptosis [20,26]. MSCs express IL-10, which is an anti-inflammatory and immunoregulatory cytokine. Furthermore, they produce IL-6 and IL-8, which are known to be associated with MSC tissue repair potential [148]. Subsequently, MSCs control the inflammatory state as evidence of the reduced expression of proinflammatory cytokines such as TNF-α, IL-1β, IL-6, and CRP [140]. Then, the STAT6 pathway is activated by IL-4, which then stimulates the MSCs to secrete TGF-β. This promotes the development of CD8+ T cells and Treg cells while suppressing the Th1 [149,150,151,152,153,154]. Moreover, MSC-secreted TGF-β has a role in macrophage polarization towards the M2 phenotype. These M2 macrophages stimulate the expression of IL-10, which alleviates inflammation. The macrophage phagocytic ability is also enhanced by TGF-β through Akt-FoxO1 pathway [36,119]. Table 2 shows the list of potential markers involved in inflammaging, which may be useful to determine the efficacy of MSC therapy.

The study of MSC effects on the immune system is largely focused on T cells rather than B cells, as its effects are more prominent in the former. Rosado et al. suggested that the prerequisite of MSCs to exert effects on B cells is a functional T cell population. Cell-to-cell contact between MSCs and T cells inhibit the proliferation and antibody production of B cells, which in turn, may aid in the management of autoimmune conditions and graft rejections [139]. Moreover, Lee et al. noted that the xenogeneic transplantation of human MSCs (hMSCs) in SLE mice models only inhibited the T cells but not the B cells. However, hMSCs that are primed with IFN-γ have increased CXCL10 and IDO expression, which effectively attracts B cells for contact inhibition [140].

In a study by Shin et al., they found that adipose tissue-derived MSCs (AT-MSCs) treatment successfully prevented the ill-effects of sepsis by mitigating the systemic inflammation and multi-organ damage. They observed the drop in pro-inflammatory markers namely IL-6 and TNF-α and reduced damage in kidney, lungs, and liver [35]. During the treatment with MSCs, there is an increased expression in inflammatory cytokines including IL-1α, IL-1β, and IL-6. It is important to note that this increase is not associated with the severity of inflammation, but it is to prime the MSCs for a sustained immunosuppression [148].

The mechanism of action of MSCs on the immune system is not constitutively inhibitory, but is acquired after exposure to the inflammatory environment with IFN-γ. IFN-γ is one of the cytokines released by T cytotoxic cells during inflammation. Therefore, in Th17 centered inflammatory response, MSC treatment would require the addition of Treg to successfully regulate the inflammation [140,172]. Lim et al. found that combination of MSCs and Treg has shown promising results in IFN-γ knockout mice with reduced inflammation and IL-7 production [172]. Additionally, Fan et al. divulged that the IFN-γ stimulation could also induce a higher expression of galectin-9 (Gal-9) in the umbilical cord-derived MSCs (UC-MSCs) through the signal transducer and activator of transcription (STAT) and c-Jun N-terminal kinase (JNK) signaling pathways. Gal-9 is one of the constitutively expressed immunomodulatory components of MSCs, which acts by suppressing CD4+ T helper cells (Th1 and Th17) and CD8+ T cytotoxic cells and regulates the suppressive activity of Treg. Even so, when Gal-9 production is inhibited, MSCs could still exert its immunosuppressive function through paracrine manner [172]. Roux et al. also observed a significant reduction in the population of both CD4+ and CD8+ T lymphocytes post-treatment with human iPSC-derived MSCs. The immunosuppression on T cells by MSCs was further substantiated with the increased expression of LAG3 and CTLA4, and cytokines including IL-10, TGF-β, and LIF [148]. Li et al. observed a significant increase in CXCR3+ Tregs in the lungs and lymphoid tissues post-MSC infusion. MSCs also increased the production of CXCL9 and CXCL10 produced by lung phagocytes which mediate the recruitment of Tregs [34].

Anderson et al.’s experiment on mice has also shown that murine AT-MSCs reduced the severity of experimental autoimmune encephalomyelitis (EAE) in mice. It is achievable due to the inhibition of the autoimmune T cell response with no increase in foxp3 Tregs. Moreover, MSCs inhibited the maturation of DCs in vitro via COX-1/2 activity and also lowered the amount of activated DCs in the lymph nodes of EAE mice [173]. DCs from the older adults have increased reactivity to self-antigen, hence their constantly activated state produces proinflammatory cytokines and stimulates the proliferation of T cells [174]. Through the inhibition of DC maturation, the inflammatory state of EAE was managed. Moreover, a study by Liu et al. transplanting human UC-MSCs into mice model showed significant improvements in the EAE pathogenesis in which the transplantation stimulated spinal cord remyelination and induced a shift of Th1 to Th2 [137]. Another study by Donders et al. using Wharton’s jelly-derived MSCs (WJ-MSCs) also found reduction in signs and severity of EAE in rats. Nevertheless, they found that the ameliorating effects of MSCs were only temporary, and the transplanted rats will clinically deteriorate again. Although repeated dosages of MSCs were administered, the disease pathogenesis of EAE did not improve [134]. This contradicting data calls for more research data on the extent of MSC regenerative capability in clinical use. Table 3 shows the effect of MSC on the immune system in human clinical studies.

All the above findings also fortify the concept that MSCs might not be a permanent solution to restore a healthy cell population. MSCs may have been seen as effective in past studies due to their paracrine effects but not cell replacement. This may explain the relatively fast drop in the inflammatory state when MSC therapy commences. Fan et al. noted that transplanted MSCs do not retain its population over time. Yet, the expression of Gal-9 continues to increase post-therapy, suggesting that a certain degree of immunosuppression can persist [172]. Li et al. postulated that the therapeutic protection of MSCs lasts more than 14 days whereas Donders et al. only observed the therapeutic effects for a week [34,134]. Additionally, Chin et al. continued to observe an increased level of anti-inflammatory cytokine IL-1RA in subjects from baseline up until 6 months post-MSC transfusion. However, note that the subjects were healthy and middle-aged which may contribute to the relatively long effectiveness of the treatment [176]. A possible solution to the limitation of MSC therapy is to find ways to sustain the survival of transplanted MSCs and increase the cell homing to the target sites to prolong the therapeutic effects.

### 5.2. Translational Application of MSCs

Bone marrow-derived MSCs (BM-MSCs) were the default source of MSCs. Nonetheless, the highly invasive procurement procedure, low cell yield (0.001–0.01% of bone marrow mononuclear cells) and multipotency that diminishes with donor age encouraged studies to be conducted on other sources of MSCs. Peripheral blood-derived MSCs (PB-MSCs) mobilized by the G-CSF are identical to BM-MSCs, but are more easily procured. However, both BM-MSCs and PB-MSCs have longer doubling time compared to MSCs from other sources [178]. PB-MSCs have been reported to possess the highest immunosuppressive capability among PB-MSCs, UC-MSCs, AT-MSCs and BM-MSCs [26]. However, contradictory results have been reported in others studies [144]. AT-MSCs can be obtained easily as surgical waste and lipo-aspirates at a high concentration up to 3% whereas UC-MSCs has the highest degree of multipotency than BM-MSCs and AT-MSCs [26].

To date, there is no definite evidence that suggests the best source of MSCs for clinical use. The sources are classified into adult and neonatal tissue derived, which have their advantages and disadvantages depending on its use. The heterogeneity among MSCs from different sources and the differences in cell treatment protocol make it impossible for direct comparison. Naturally, MSCs derived from perinatal tissues have stronger immunomodulatory properties compared to the aged source. UC-MSCs and WJ-MSCs are non-senescent, highly proliferative and with potent differentiation potential [26,179]. In application regarding immunosenescence, declining cellular functions and chronic sub-clinical inflammation are of concern in an aging person. Hence, allogenic UC-MSCs and WJ-MSCs seem more suited to be used in this subject matter. Nevertheless, the plasticity of UC-MSCs relies on the metabolic condition of the mother during pregnancy. UC-MSCs collected from gestational diabetes mellitus mothers displayed earlier cellular senescence and decreased cell growth [180]. Therefore, UC-MSCs should be sourced from healthy mothers to ensure a high biological quality of stem cells is obtained for clinical use. Abolhasani et al. also reported that the gestational age and in vitro expansion can influence the immunomodulatory properties of UC-MSCs [181].

Next, the optimal method of administering MSCs has yet to be determined. The MSCs can be introduced into the body locally or systematically. Local administration of MSCs targeted to the injury site and produced rapid results. However, there is a risk of cell death and bleeding at the site of application [182]. The systemic administration including intraperitoneal (IP), intravascular (IV), subcutaneous (SC), and intramuscular (IM) delivery have varying cell fate and therapeutic efficacy. Castelo-Branco et al. found that the IP method produced better homing and inflammation suppression than IV [33]. On the contrary, Gonçalves et al. contended that the IV administration of MSCs was more effective than IP method in the treatment of colitis as IV administration managed to stimulate a higher level of immunosuppression [183]. However, MSCs administered through the IV route tend to become entrapped in the lungs, with only 10% of the transplanted cells accumulate at the site of damage [34,182,184]. Roux et al. stated the preference of IP over IV as to avoid the risk of pulmonary embolization which may lead to the surge of an anti-inflammatory protein known as TSG6 [148]. IM injection is another possible route of MSC delivery which is advocated by Braid et al. for producing the longest cell retention time in the host body when compared to IV, IP, and SC, which was more than 100 days. Both IM and SC implantation sites also retained most of the MSCs, which shows a potential for controlled MSC dosage [184]. Furthermore, IM is less invasive than IV. Nonetheless, the research data on effects of IM administration of MSCs on the immune system is inadequate compared to the more established IV method.

Ueda et al. injected MSCs contained in collagen scaffold to the dorsum part of mice which considerably prolonged the retention of MSCs at the transplantation site for at least 2 weeks. The collagen scaffold acted as a reservoir for the exogenous MSCs and preserved the self-renewal, multipotency, and homing functions of MSCs. Furthermore, the formation of aggregates, which commonly occurs with IV administration can be avoided [182].

Ebrahim et al. compared the efficacy of standard treatment using antileukotriene drug (Montelukast) versus MSCs in the treatment of allergic rhinitis. MSCs exert immunomodulatory effects on the adaptive immunity by shifting the TH1/TH2 balance through T cell suppression and production of Treg. Rats treated with MSCs showed significant improvement in the allergic and inflammatory response and less damage to the nasal epithelium, which is superior compared the rats treated with antileukotriene drug [185]. El-Gendy et al. compared AT-MSCs with etanercept in terms of preventive and therapeutic efficacy in rheumatoid arthritis. Etanercept is an anti-inflammatory drug commonly prescribed for rheumatoid arthritis and other inflammatory conditions. The results of rats treated with both groups are comparable in terms of suppression of clinical signs, less severity of joint deformity, and modulation of immune responses. The etanercept group showed the lowest TNF-α level but the AT-MSCs group had significantly higher levels of Treg cells and IL-10 [186]. These results showed the promising prospect of MSCs to substitute the current prescription in improving inflammatory conditions.

Golpanian et al. and Tompkins et al. conducted the phase I and phase II clinical trials in aged patients by administering different doses of allogeneic MSCs through the IV route. The studies monitor the adverse effects as well as the patients’ physical performances and TNF-α level for six months. Both studies demonstrated that 100 million allogenic MSCs is the most optimum dosage in frail patients which produced significant improvements in both physical and inflammaging condition, noting reduced circulating TNF-α level. Safety of IV administration of allogeneic MSCs is also demonstrated when treatment emergent-serious adverse events are absent in the treated patients [138,175].

Zheng et al. plotted an extensive immune cell landscape in aging and COVID-19. In general, COVID-19 patients who are elderly have shown immune cell polarization and upregulation of inflammatory genes. There is a decrease in TCR and BCR diversity and an increase in clonality of effector, cytotoxic, and exhausted T cells. The NK cells and B cells have decreased antigen-presenting ability due to the upregulated inflammaging. Besides, the phenotype of mononuclear cells involved are inflammatory and persist at a higher ratio than the T cells. To add insult to the injury, aging also increases the expression of the COVID-19 susceptibility genes. Unsurprisingly, the elderly patients have lowered threshold of triggering cytokine storms and lymphopenia, which result in higher mortality from the infection [86]. MSC has been actively studied for COVID-19 treatment. Along with the urgency of the COVID-19 pandemic, numerous clinical trials have been proposed urgently to suggest MSC as an endogenous biological intervention to reduce the severity of the disease. At the time of writing, there are 53 clinical trials registered on the https://clinicaltrials.gov/ (accessed on 20 May 2021). In February 2020, a critically ill COVID-19 patient with severe pneumonia, ARDS and multi-organ injury was treated with hUCMSCs adoptive transfer therapy. Shortly after the treatment, their haematologic parameters, immune cell count, blood chemistry and clinical presentation of pneumonia vastly improved in a short time. After 8 days, the patient was discharged from the intensive care unit (ICU). Even though this study had only documented the recovery of one patient, it is remarkable that MSC may hold such premises [187]. Haberle et al. had also seen great improvement in their MSC treatment group. At the start of the study, the selected MSC treatment group had more severe COVID-19 ARDS than the control group, as indicated by the higher Murray score for lung injury. Finally, the MSC group showed significantly better pulmonary function and reduced inflammatory cells at discharge when compared to the control group [188]. Hashemian et al. also reported a significant decrease in the major inflammatory biomarkers (CRP, IL-6, IL-8, and TNF-α) and a significant improvement in the opacities of the lung CT scans after the MSC infusions [177]. 

## 6. Limitations and Prospects

The issue with stem cell therapy is the directed differentiation of the transplanted cells into functional mature tissue. Currently, the data on the effects of MSC on the immune system are mostly collected from animal studies and extrapolated to human use. Laparra et al. stated that there are significant differences in the immune compartment of adipose tissue of human and mice in which human carry a more inflammatory steady-state profile than mice. These differences may affect the accountability of the intended translational studies [189]. It is possible to derive thymic epithelial cells from embryonic stem cells or iPSCs to develop humanized mouse models that represent a higher resemblance to human immunity [43]. Even then, the accumulated data from current studies are non-specific to inflammation related to aging itself. Furthermore, there are discrepancies in results observed in different animal models and experimental conditions. Table 4 summarizes the in vivo data reporting the effects of MSC transplantation on the immune system in the pathological disease model.

MSC therapy includes the injection of a large number of cells. Thus, it may pose safety issues and side effects to the patient. Koch et al. determined that the exosomes extracted from MSCs can circumvent the safety concern and still exert immunomodulatory functions by reducing the NK cell number and TNF-α transcription [193]. Mead et al. also observed promising results in using MSC-derived exosomes which exerted neuroprotective effects on human stem cell-derived retinal ganglion cells [194]. Furthermore, the authors also found that priming of MSCs with TNF-α prior exosome collection enhances the neuroprotective effects.

To date, the optimum dosage of MSCs for transplantation is still undefined. Even though studies have reported that the use of dosage as high as 1200 million cells is safe; however, a higher dose does not indicate higher therapeutic efficacy as several studies have reported better clinical outcomes in the lower dosage group. In fact, the minimum dosage that is effective was found to range between 100 and 150 million cells, while doses higher than 200 million were found to be less or not effective [195]. In a phase II clinical study, Tompkins et al. discovered that only the 200 million group managed to reduce CD8, thus reinstituting the previously aging-reduced CD4/CD8 ratio. Nevertheless, there are no significant therapeutic differences between 100 million and 200 million cells in the other tested parameters which brought to the question on why the therapeutic effects are limited to 100 million even though the dosage was doubled [138]. Another recent clinical trial concluded that administration of 130 million cells is more effective than the lower dosage of 65 million cells in producing immunomodulatory effects in healthy patients [176]. Therefore, it is postulated that a specific range of cell number is needed to exert the therapeutic effects and administration of excessive cells does not provide additional benefits. In the future, studies should be conducted to validate the minimum effective dose of MSCs that can effectively ameliorate immune system aging.

Current management of acute respiratory distress syndrome (ARDS) is focused on supportive treatment techniques such as intubated ventilator-assisted breathing, the morbidity and mortality of ARDS remain high [196]. MSCs have been widely investigated as a potential therapy for ARDS. To date, there have been four completed and five ongoing clinical trials involving the treatment of MSC in ARDS on http://clinicaltrials.gov (accessed on 20 May 2021). Note that the immunomodulatory mechanism of MSCs is still indefinite and their potential adverse effects should not be overlooked. Islam et al.’s crucial findings discouraged the administration of MSC in a disease state unless a suitable immunologic profile is achieved. They found that MSCs could be stimulated by plasma of ARDS patient, which will lead to a possible phenotypic shift (decreased CD105 and CD90 expression) and the exacerbation of ARDS (increased levels of IL-6, fibronectin, and cytotoxicity). This study also highlighted a need to ensure that the proper microenvironment indication is met before MSC therapy and that the approach to certain disease condition should be precise. It is unsure that this applies to the frailty condition [192]. Note that the age of the COVID-19 patients have an impact on the severity of the disease as discussed earlier, with one of the severe manifestation of the disease being ARDS. However, the clinical trials did not explore on the microenvironment of prior to the administration of MSC treatment.

There are two contrasting viewpoints on the role of TNF-α in MSC therapy. TNF-α is an inflammatory cytokine released by monocytes during inflammatory disease pathogenesis. Reduction of TNF-α using TNF-α inhibitors, such as etanercept, can reduce the inflammation. However, MSCs may not be strongly activated for immunosuppression when TNF-α inhibitors are introduced before MSC transplantation. Gendy et al. did not observe any protective functions from MSCs which suggested that MSC immunosuppressive response is activated by inflammatory signals, such as TNF-α [186]. Mead et al. observed that priming MSCs with TNF-α further enhanced the neuroprotective effect of the secreted exosomes on retinal ganglion cells compared to the exosomes secreted by the unprimed cells [194]. On the other hand, Senyuk et al. found that the blockade of TNF-α using etanercept before and after transplantation of HSCs facilitates long-term engraftment. In in vitro culture, TNF-α was found to downregulate the genes that regulate pluripotency such as NANOG, SOX2, and OCT4. TNF-α also caused the epigenetic dysregulation of CD34+ which reduced the transplantation capacity [197]. The role of TNF-α priming or blockade paired with stem cell transplantation may produce significant data for curative treatment of inflammaging. Hence, a comparative study should be done to determine the better therapeutic approach and the condition for administration.

Priming can modulate the MSC immunomodulatory functions. Vega-Letter et al. primed MSCs using poly(I:C) to stimulate expression of TLR3, which enhances its immunosuppressive capacity. TLR3-MSCs demonstrated more potent inhibition of Th1 and Th17 proliferation in vitro. The authors also found that MSCs pretreated with lipopolysaccharide (LPS) showed higher expression of TLR4 and produced a pro-inflammatory response that diminished inhibition of Th1 and Th17 proliferation [198]. In another study, Liu et al. compared the therapeutic effects of TLR2- and IFN-γ-stimulated MSCs with TLR4- and IFN-γ-stimulated MSCs in treating schistosome-infected mice. TLR4-IFN-γ-MSCs induced Th1 to restore Th1/Th2 balance and alleviate liver fibrosis. TLR2-IFN-γ-MSCs not only increased Th1 more intensely but also inhibited Th2 activity, which led to excessive inflammatory response [159]. The primed MSCs with more potent immunosuppressive capacity may reduce the dosage required and provide clinical benefits that sustain for a longer period. Additionally, it would be interesting to see if primed MSCs have longer survival period post-transplantation.

On top of the immunology profiles, the effects of MSC therapy on metabolomic and lipidomic profiles that are closely related to immune function of aged individuals also should be studied in the future. The lipidomic markers of centenarians showed relatively functional antioxidant capability and lower lipid peroxidation inflammatory state. Healthy older adults have significantly higher level of phenylalanine and reduced level of glycerophosphocholine. Phenylalanine inhibits the NF-κB pathway, which is a pro-inflammatory signaling pathway that induces cytokine production, whereas glycerophosphocholine is a lipid biomarker of cell senescence [199,200,201,202]. The metabolomic profile of centenarians also presented a remodeled mechanism of cellular detoxification through heightened cytochrome P450 enzyme activity and arachidonic acid synthesis which displayed both pro-inflammatory and anti-inflammatory characteristics [202,203]. It will be interesting if future studies can examine the effects of MSCs on these metabolomic and lipidomic biomarkers as indicators of immune function modulation. The measurement of serum albumin and globulin also helps to reflect the immunity status of a person as a decreased albumin/globulin ratio indicates a higher risk in developing chronic diseases [204]. Along with that, it is also necessary to validate the inflammatory markers that appear rather consistently to reliably evaluate the clinical outcomes of MSC therapy.

Allogeneic hematopoietic stem cell transplantation (allo-HSCT) is a choice of treatment for many hematological malignancies and autoimmune diseases. It involves the replacement of defective hematopoietic cells with long term repopulating cells from a donor. A related donor with matching HLA is preferable for treatment but it is seldom available. Administering an allogeneic source of HSC is viable. Nevertheless, this puts the recipient at risk of poor reconstitution of the adaptive immune system or worse- graft rejection, graft-versus-host disease (GVHD) and graft-versus-tumor (GVT) [205]. In GVHD, the thymus is vulnerable to attacks by the alloreactive T cells, which hamper the reconstitution of a healthy T cell population [206,207]. 

There are three options of conditioning prior to HSCT- myeloablative (MA), non-myeloablative (NMA), and reduced intensity (RI). MA and RI are frequently compared on their efficacy and safety aspects. Bacigalupo et al. defined MA conditioning HSCT as a treatment that induces an irreversible cytopenia and necessitates stem cell support, whereas RI may or may not cause irreversible cytopenia and the patient can be given stem cell support [208]. RI is recommended to be used in elderly patients as it is associated with less non-relapse mortality (NRM) even in high-risk patients when compared to MA [209,210,211]. Aoki et al. reported that the advanced age of recipients does not contribute as a contraindication to RI conditioning allo-HSCT [211]. 

The efficacy of HSCT is highly dependent on the condition of the thymus where the maturation of T cell takes place. The T cell repertoire generated in the elderly is usually less diverse or delayed due to thymic involution [212,213]. The bone marrow stromal cells are also damaged by the conditioning pre-HSCT, which results in a limited B cell lymphopoiesis [214]. There are strategies developed to circumvent these faults, such as modified donor lymphocyte infusions from the same haploidentical donor or using a suicide gene, i.e., inducible Caspase 9 (iCas9) [212]. Furthermore, HSCT can be supported with MSC. A healthy and abundant MSC population is essential in differentiating into an environmental niche required to support the HSC and its lineages. A co-infusion of HSC and MSC promotes engraftment and reduce the risk of developing GVHD without exacerbating the NRM [32,215]. According to Abbuehl et al., co-infusion of HSC with BMSC doubles the number of functional, transplanted HSCs, while reducing the adverse effects of HSCT including neutropenia and humoral immunodeficiency [214].

## 7. Conclusions

Immunosenescence is an inevitable phenomenon that involves the remodeling of the immune system with age. This complex interaction between the age-accumulated insults, aged HSCs bias to myeloid cells, and both the innate and adaptive immune system results in a chronic, subclinical systemic inflammation termed as ‘inflammaging’. The individuals over 65 years old have increased risk of infection, cancer, higher morbidity, and mortality of disease, and reduced vaccine efficacy. Currently, there are no effective countermeasures available to ameliorate immunosenescence. MSC therapy is a promising modality to rejuvenate the aged immune system. As of now, studies have shown that MSCs can safely reduce the inflammatory markers, restore the T cell repertoire, and improve the histopathology of inflammatory disease.

## Figures and Tables

**Table 1:** The functional changes of immune cells in immunosenescence and the current evidence of MSC that improved the function of the aged cells. (‘↓’ = decrease; ‘↑’ = increase; ‘-‘ = no change).

Cell	Functional Changes in Immunosenescence	Implications	Current Evidence of MSC Research
Innate immune cells			
Monocyte/Macrophage 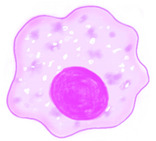	↑ polarization from M1 to M2 phenotype [80] ↑ proinflammatory cytokines [80] ↑ CD38 [115] ↓ antioxidant [116] ↓ NAD+ [117]	↓ removal of senescent cells [80] ↓ TLR expression and function [118] ↓ phagocytosis [36,116]	Induced polarization of M2 to M1 [36,119] Enhanced the macrophage phagocytic ability [36] Increased the expression of anti-inflammatory factors [119]
Neutrophil 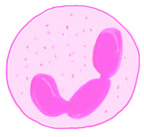	↑ TLR4 [84] ↑ adhesion molecules [95] ↑ intracellular calcium [93] ↑ NET [84] ↑ CXCR4 [84] ↓ sensitivity to G-CSF [120] ↓ intracellular ROS ↓ CD44 [94] ↓ CD62L [84]	↑ aberrant migration [121] ↓ clearance [94] ↓ phagocytosis [122]	Reduced expression of cytokine-induced neutrophil chemoattractant-1 and IL-6 [123] Reduced neutrophil infiltration in respiratory infection [34] Enhanced the neutrophil phagocytic ability [124] Suppressed NET formation [125]
NK cell 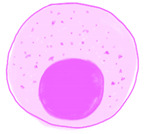	↑/- KIR [126] ↑ CD56^DIM^ NK [97,127] ↓ CD94 [126] ↓ NKp30 or NKp46 expression [127] ↓/- perforin-mediated NKCC [126] ↓ CXCR1 [126]	↓/- Lytic activity [126] ↓ production of cytokines and chemokines [97] ↓ clearance [126] ↓ reduced anti-microbial function [126]	Decreased in IFN-γ production [128] Inhibited NKCC [129]
Adaptive immune cells			
T cells 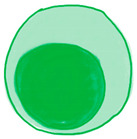	↑ Treg [88,106] ↓ naïve cells [113,130] ↓ diversity in TCR [130,131] ↓ CD28 expression [104]	↑ proinflammatory cytokines [131] ↑ autoimmunity [7] ↑ risk of infection [3,4] ↓ clearance [131]	Decreased the secretion of IFN-γ by Th1 [128] Increased secretion of IL-4 by Th2 [128] Increased proliferation of Treg [34,128] Inhibited T cell proliferation [128,132,133,134,135,136] Induced phenotypic shift of Th1 to Th2 [137] Decreased CD8 population [138]
B cells 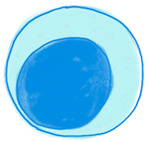	↑ Intracellular TNF-α [111] ↑ BCR clonality [100] ↓ decreased AID expression [100,102]	↓ response to novel antigen [100] ↓ somatic hypermutation [100] ↓ class-switch recombination [100]	Inhibited B cell proliferation [139,140] Reduced IL-10 production [139]

NAD—Nicotinamide adenine dinucleotide, TLR—toll-like receptor, NET—neutrophil extracellular traps, G-CSF—Granulocyte colony-stimulating factor, ROS—reactive oxygen species, KIR—Killer-cell immunoglobulin-like receptor, NKp—NK cell precursor, CXCR—CX-chemokine receptor, NKCC—Natural killer cell cytotoxicity, IFN-γ—Interferon-gamma, Treg—T regulatory cell, TCR—T cell receptor, Th—T helper, TNF-α—Tumor necrosis factor α, BCR—B cell receptor AID—activation-induced cytidine deaminase, IL—interleukin.

**Table 2:** The potential ‘inflammaging markers’ related to inflammatory diseases and aging. These markers may be used to validate the efficacy of MSC treatment. (‘↓’ = decrease; ‘↑’ = increase; ‘-‘ = no change).

Potential ‘Inflammaging Markers’	Status in Inflammaging	References
IGF-1	↓	[17,155,156]
CD4+ T cells	↓	[19,40,81,98]
CD28+ T cells	↓	[11,157,158]
CD19+ B cells	↓	[88,114]
IL-10	↓/-	[2,35,39,50]
TGF-β	↓	[33,156,159,160]
IL-2	-	[161]
IFN-γ	↑	[161,162]
TNF-α	↑	[161,163,164]
IL-6	↑	[15,36,156,165,166]
WBC	↑	[17]
CD8+ T cells	↑	[19,40,81,98,103,157,167]
CD56+ NK cells	↑	[86,96,97,103,126,168]
IL-1β	↑/-	[36,164]
IL-15	↑	[164]
IL-18	↑	[164]
CD68	↑	[163]
MCP-1	↑	[163]
IL-17	↑	[34]
IL-8 (CXCL8)	↑	[11,86]
CXCL10	↑	[169,170]
CCL2	↑	[170,171]

**Table 3:** A summary of clinical studies of MSC effects on the immune system from 2017–2021.

References	Human Subjects	MSC and Dosage	Results (Related to Immune Cells and Inflammatory Markers)	Additional Notes
Golpanian et al. (2017) [175]	An average age of 78.4 ± 4.7 years and Clinical Frailty Score of 4–6	Group 1 = 20 × 106 allo-hBM-MSCs, IV injection	Group 2 and Group 3 showed significant decrease in TNF-α, whereas Group 1 showed moderate reduction.	100 × 10^6^ cells is the optimal dose level.
		Group 2 = 100 × 106 allo-hBM-MSCs, IV injection		
			No significant changes were seen in CRP, IL-6, fibrinogen, D-dimer, and white blood cell counts.	No additional benefit or loss of effect when 200 × 10^6^ cell dose was used.
		Group 3 = 200 × 106 allo-hBM-MSCs, IV injection		
Tompkins et al. (2017) [138]	Age ≥60 and ≤95 years with Clinical Frailty Score of 4–7	Group 1 = 100 × 10^6^ allo-hBM-MSCs, IV injection	Decreased serum TNF-α levels in Group 1.	No therapy-related side effects occurred.
			Decreased B cell intracellular TNF-α in both Group 1 and Group 2.	
			Decreased early CD 69 and late activated CD25 T cells in both Group 1 and Group 2.	
			Decreased CD8 in Group 2.	
		Group 2 = 200 × 10^6^ allo-hBM-MSCs, IV injection	No changes in CD4 in both Group 1 and Group 2.	
			CD4/CD8 ratio increased in Group 2.	
			No significant changes noted in IL-6, CRP, D-dimer, CBC, and fibrinogen in both Group 1 and Group 2.	
Chin et al. (2020) [176]	Healthy, non-frail subjects with mean age of 55 ± 13 years	Group 1: 65 × 10^6^ allo-hUC-MSCs, IV injection	In Group 1, no significant changes were noted in the serum levels of IL-10, IL-1RA, IL-6, PGE2, and TNF-α.	No therapy-related side effects occurred.
			In Group 2, the serum IL-1RA level was significantly increased for at least 6 months post-infusion.	
			The serum IL-6 level throughout the 6 months monitoring period was higher in Group 2 than in Group 1.	The immunoglobulin E (IgE) level remained low within the normal range which indicated that there were no hypersensitivity reaction post-infusion.
		Group 2: 130 × 10^6^ allo-hUC-MSCs, IV injection	The serum TNF-α level was significantly lower at day 2 in Group 2 than Group 1.	
			Both Group 1 and Group 2 observed a significant increase in C-reactive protein at day 2 post-infusion, which then dropped continuously over 6 months.	No significant changes in total white cell count or its subfractions post-infusion.
			The albumin/globulin ratio was higher in Group 2 than in Group 1 at 6 months.	No significant changes in the lung function tests (FEV1 and FEV1/FVC levels) post-infusion.
				No significant changes in the growth factors (VEGF, TGF-β, and HGF) level post-infusion.
Hashemian et al. (2021) [177]	11 patients diagnosed with COVID-19-induced ARDS who were admitted to the intensive care unit, age range was 42–66 years old	3 × IV injections (200 × 10^6^ cells) every other day for a total of 600 × 10^6^ hUC-MSCs (6 cases) or PL-MSCs (5 cases).	Significant reductions in serum levels of TNF-α, IL-8 and CRP were seen in all six survivors.	All six survivors were well with no complaints of dyspnea on day 60 post-infusion.
			IL-6 levels decreased in five patients.	Radiological parameters of the lung CT scans showed great signs of recovery.
			IFN-γ levels decreased in four patients.	Four patients who had signs of multi-organ failure or sepsis died in average 10 days after the first MSC infusion.
			IL-4 and IL- 10 levels increased in four cases, but the differences were not statistically significant.	

FEV1—forced expiratory volume in one second, FVC—forced vital capacity, COVID-19—Coronavirus disease 2019, ARDS—Acute Respiratory Distress Syndrome, PL-MSCs—placental MSCs, CT—computed tomography.

**Table 4:** A summary of in vivo studies of MSC effects on the pathological immune system from 2012–2020.

References	In Vivo Model	MSC and Dosage	Results (Related to Immune Cells and Inflammatory Markers)	Additional Notes
Shin et al. (2012) [35]	Male Sprague–Dawley rats (Induced endotoxemia)	2 × 10^6^ hAT-MSCs in 100 μL saline solution, IV tail vein injection	Decreased the pulmonary IL-6 level.	Endotoxemia animal model does not completely reflect septic conditions in human.
			Decreased serum and pulmonary TNF-a levels.	Improved multi-organ dysfunction induced by LPS.
			No changes in pulmonary IL-10.	
Gosemann et al. (2012) [190]	Male C3H/HeN wild type mice (Induced endotoxemia)	1 × 10^6^ human amniotic fluid-MSCs in 700 μL PBS, IP injection	No significant difference in serum IL-6, TNF-α, MCP-1, IL-10 and TGF-β.	-
			Increased serum IL-2.	
			Increased percentage of CD4 + CD25+ lymphocytes expressing Foxp3+ (Tregs).	
			Decreased pulmonary neutrophil infiltration.	
Donders et al. (2015) [134]	Female dark agouti rats (8 weeks old, Induced EAE)	2 × 10^6^ hWJ-MSCs in 500 μL saline solution, IV tail vein injection	In contact co-culture, suppressed polyclonal-induced T cell proliferation and IFN-γ production.	EAE symptoms improved.
			Modulated DC differentiation and maturation in the presence of LPS.	Therapeutic effect lasted for about 1 week.
Lee et al. (2015) [191]	BALB/c (H-2d) mice (8–10 weeks old) as BM-MSCs and spleen cells recipient C57BL/6-tg (CAG-EGFP; H-2b) mice as BM-MSCs and spleen cells donor	5 × 10^6^ allo-mBM-MSCs and 5 × 10^6^ spleen cells, IV injection	Decreased Th1 secretion of IFN-γ and TNF-α.	Combined cell therapy improved the clinical outcomes in the murine aGVHD model.
			No changes in Th2 secretion of IL-4.	
			Decreased IL-17 within CD4+ T cells.	
			Increased CD4+ CD25+ cells.	
			Increased Foxp3+ T reg cells.	
Islam et al. (2019) [192]	Male C57BL/6 mice (14–16 weeks old, HCl-induced lung injury model)	0.5 × 10^6^ cells/50 mL phosphate-buffered saline (PBS) through intratracheal instillation. 5 minutes later, 0.5 × 10^6^ cells/100 mL PBS via tail vein IV injection	MSC administration 2 days after HCl stimulation increased fibrotic markers including TGF-β1, FN in BALF, and fibrinogen.	
			On the contrary, MSC is protective when administered 14 days after HCl stimulation.	
			After correction of lung microenvironment using lentivirus carrying human GPx-1, MSC administered 2 days after HCl stimulation showed protective effect (lowered Ashcroft score and reduction in the level of FN in BALF.	
			After stimulation of MSC and ARDS patient plasma, MSC phenotype shifted (decreased CD105 and CD90) at 5 days.	
El-Gendy et al. (2020) [186]	Female Sprague–Dawley rats (6–8 weeks old, CIA)	1 × 10^6^ hAT-MSCs in 100 μL PBS, IP injection	Low percentage of autoreactive CD4+ and CD8+ T cells in spleen.	Suppressed clinical signs and histopathology of CIA.
			High percentage of FOX3P+ CD25+ Treg.	
			Insignificantly reduced serum TNF-α and anti-CII antibody levels.	
			Increased the levels of IL-10.	
Lee et al. (2020) [140]	Female Lupus-Prone MRL/Faslpr Mice (12 weeks)	4 × 10^5^ hBM-MSCs in PBS, IV injection	Decreased the expression levels of all examined cytokines including IL-2, IL-4, IL-6, IL-10, IFN-α, IFN-β, IFN-γ, IL-1β, TNF-α.	Naïve hBM-MSCs can only inhibit T cells.
			Inhibited both T and B cells in the presence of IFN-γ.	
			Inhibited B cells through IDO and CXCL10-dependent manner.	
Li et al. (2020) [34]	Female C57BL/6 mice (7–8 weeks old, pristane induced lupus)	1 × 10^6^ hUC-MSCs, IV injection	Increased TGF-β levels.	Reduced the severity of respiratory infection with improved bacterial elimination and increasing Tregs.
			Inhibited CCL2 (MCP- 1), TNF-a, IL-6, IL-1b and IL-17A after 24 h of infection.	
			Increased Treg with high expression of CXCR3 on day 3.	
			Induced production of CXCL9 and CXCL10 by lung phagocytes.	

LPS—lipopolysaccharide, EAE—experimental autoimmune encephalitis, aGVHD—acute graft-versus-host-disease, CRP—C-reactive protein, CBC—complete blood count, CIA—collagen-induced arthritis, HCl—hydrochloric acid, BALF—bronchoalveolar lavage fluid.

## Data Availability

Not applicable.
